# Hand Warmers: A Cost-Effective Solution to Accelerate Oxygen Depletion During Hermetic Storage

**DOI:** 10.3390/foods14040548

**Published:** 2025-02-07

**Authors:** Wenbo Li, John Stephen Yaninek, Kingsly Ambrose, Dieudonne Baributsa

**Affiliations:** 1 Department of Entomology, Purdue University, West Lafayette, IN 47907, USA; li2793@purdue.edu (W.L.); yaninek@purdue.edu (J.S.Y.); 2 Department of Agricultural and Biological Engineering, Purdue University, West Lafayette, IN 47907, USA; rambrose@purdue.edu

**Keywords:** airtight storage, postharvest innovation, oxygen scavenger, grain preservation

## Abstract

Postharvest grain losses often result from insect infestations. Hermetic storage creates airtight conditions that limit insect survival. However, oxygen depletion can be slow during hermetic storage, leading to a loss of grain quality and market value. Oxygen scavengers offer a solution to accelerate oxygen depletion. This study evaluated hand warmers as a cost-effective alternative to commercial oxygen scavengers. Experiments in sealed empty 4-gallon glass jars with 10-h hand warmers depleted oxygen faster and more cost-effectively than those with 2000 cc Oxy-Sorb oxygen absorbers. One hand warmer depleted similar amounts of oxygen as two Oxy-Sorb oxygen absorbers and reached the 5% threshold for pest suppression in 48 h. A follow-up study found that oxygen levels in empty 4-gallon jars dropped faster than in grain-filled 25-kg hermetic bags, with jars containing two or three hand warmers reaching the 5% threshold in the first 6 h. Temperature remained constant regardless of the number of hand warmers. At the same time, the relative humidity rose in empty jars but stayed stable in grain-filled hermetic bags, with no effect on grain quality. Hand warmers can potentially serve as cost-effective alternatives to commercial oxygen scavengers in hermetic storage.

## 1. Introduction

Postharvest losses pose a significant challenge globally as stored grain is vulnerable to damage, contaminants, and nutrient loss due to various biotic and abiotic factors. Biotic factors include insects, rodents, and fungi, while abiotic factors encompass temperature, relative humidity, and moisture content. Among the biotic factors, insects are the leading cause of losses [1,2]. Farmers often use chemical controls, such as insecticides, to eliminate insects in stored grain [3]. However, chemical controls pose health risks to consumers and applicators and contribute to environmental pollution. In developing countries, where awareness and training on insecticide use are limited, high rates of insecticide poisoning are documented due to misuse and overuse [4,5].

Hermetic bags have become an alternative to chemical control methods in developing countries [6,7]. These bags could also benefit farmers who store specialty crops or small quantities of seeds and grain as the need for alternative pest control methods grows with the phasing out of many chemicals [8,9]. The effectiveness of hermetic bags lies in their airtightness, which allows the metabolic activity of insects and other organisms to significantly reduce oxygen levels, inhibiting feeding and reproduction [10]. These airtight conditions not only prevent insect infestations but also limit mold growth [11,12].

While hermetic bags can effectively control insect pests, they face a few challenges. When the initial insect populations are low, (intergranular) airspace is greater, or when infested grain is stored in cooler climates, oxygen depletion may take longer to reach levels critical for halting insect development [13,14,15]. In these conditions, grain damage may still occur, as the extended time for oxygen reduction increases the risks of quantity and quality loss. A study on cassava chips demonstrated that insect metabolism alone was insufficient to reduce oxygen to lethal levels due to excess airspace within the stored product [16]. In addition, fortified products like provitamin A maize require anoxic conditions to preserve quality, which hermetic bags alone may not provide [17,18,19,20]. In the specialty markets of developed countries where pesticide use is limited and tolerance for insects in food is minimal, relying solely on natural processes during hermetic storage may be too risky. Finding a cost-effective approach to accelerate oxygen depletion could minimize insect damage and preserve product quality.

Controlled atmospheres in food packaging to deplete oxygen have been applied to preserve quality and minimize insect infestations [21]. In commercial grain storage facilities (between 50 and 2500 MT capacities), controlled atmospheres using gases such as carbon dioxide or nitrogen have been utilized to manage insect pests [22]. Other approaches, such as using soaked or germinating seeds, have been explored to deplete oxygen [14,23]. However, improvements in technology and cost efficiencies are needed to encourage broader adoption. For farmers storing small quantities, the process of displacing oxygen with gases is too complex and costly as it requires specialized equipment (e.g., pressurized tanks) and operational skills [7,24,25,26].

Oxygen-scavenging systems available as sachets, sheets, trays, pads, or labels, use inorganic (iron-based, platinum metals) or organic (ascorbic acid, enzymes, microorganisms) compounds to react with and remove oxygen, preventing oxidation in food packaging [27,28]. Most inorganic oxygen scavengers, being mainly metal-based with materials such as ferrous iron powder, are made of salt and activated carbon in various-sized packages [29,30]. Although ingredients beyond ferrous iron, salt, and activated carbon vary to meet specific needs, oxygen depletion always occurs through an oxidation reaction (Equation (1)). When exposed to air, ferrous iron oxide absorbs moisture from the environment, activating it to oxidize into hydrated iron [21].

(1) $$4Fe+3O_{2}+6H_{2}O\to 4Fe({OH)}_{3}$$

Most commercially available oxygen scavengers are designed for use in small packages, such as those for food and beverages, including bread, meat, fish, fruit, and cheese [21,31,32]. This raises questions about their suitability for depleting oxygen in hermetic bags used for grain storage. Scavengers vary in oxygen depletion capacity, typically ranging from 20 to 2000 cc (mL) [33]. Hand warmers, with the same chemical composition as many oxygen scavengers, are designed to generate heat to warm the body in cold conditions. This exothermic process, based on the oxidation of iron powder, raises temperatures between 37 and 82 °C when activated [29]. It follows the same reaction as the oxidation of iron powder-based oxygen scavengers (see Equation (1)).

Oxygen scavengers, when combined with hermetic containers, can maintain near-zero oxygen levels in food packaging and grain storage [19,34,35]. Although oxygen scavengers can create hypoxic conditions for grain storage, the efficacy of hand warmers in depleting oxygen has not been studied. This study aimed to (i) assess the performance and cost-effectiveness of hand warmers as oxygen scavengers and (ii) evaluate their impact on environmental conditions (temperature and relative humidity) and subsequent effects on grain quality (moisture content and germination rate of stored seed). We hypothesized that hand warmers would perform as well as or better than a commercial oxygen scavenger, offering a cost-effective alternative without any decrease in grain quality.

## 2. Materials and Methods

This research was conducted from Fall 2022 to Spring 2024 in the Postharvest Innovation for Crop Storage laboratory in the Department of Entomology at Purdue University (West Lafayette, IN, USA).

### 2.1. Availability and Cost of Oxygen Scavengers

Since hand warmers and Oxy-Sorb oxygen absorbers are not needed year-round and have limited availability in local stores, we assessed their cost and availability through an online survey.

### 2.2. Experimental Set-Up

The study consisted of two experiments (i) evaluating the use of hand warmers as cost-effective oxygen scavengers and (ii) assessing the effect of hand warmers on environmental conditions in hermetic storage. Oxygen scavengers were purchased online from Amazon.com, Inc. The 2000 cc Oxy-Sorb oxygen absorbers, manufactured by Dry Pak Industries, Inc. (Los Angeles, CA, USA), were the highest-capacity scavengers in the product line, while the 10 and 18-h capacity HotHands^®^ hand warmers were made by Kobayashi Healthcare International, Inc. (Dalton, GA, USA). All products used in this study were purchased on 20 November 2022, at unit prices of USD 0.70, 0.60, and 0.30 for the Oxy-Sorb oxygen absorbers and the 18- and 10-h hand warmers, respectively.

#### 2.2.1. Experiment One: Efficacy of Oxygen Scavengers

**Figure 1:**
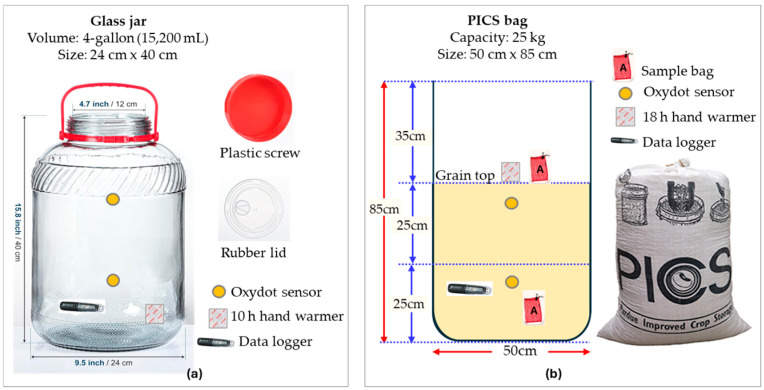
Schematic representation of experimental (**a**) 4-gallon glass jar and (**b**) 25-kg PICS bag with the modifications used in this experiment. Image (**a**) adapted from Amazon.

To assess whether hand warmers could be an effective alternative to existing oxygen scavengers, we conducted an experiment comparing their efficacy to a commercially available product, the Oxy-Sorb oxygen absorber. This experiment compared 10-h hand warmers (5.08 × 8.89 cm) to 2000 cc Oxy-Sorb oxygen absorbers (8.13 × 11.68 cm). Each treatment combined 1 or 2 units of either hand warmers or Oxy-Sorb oxygen absorbers, with 4 replicates per treatment giving a total of 16 jars. Each treatment was placed in an airtight 4-gallon (15,200 mL) glass jar (Daitouge, Qingdao, China) purchased online (Figure 1a) [36]. Each jar was sealed with a rubber airtight lid then covered with a second plastic screw lid, and a piece of parafilm was wrapped around the second lid to ensure hermeticity.

#### 2.2.2. Experiment Two: Effects of Hand Warmers on Hermetic Environmental Conditions

In the first experiment, we observed that relative humidity was accumulating inside empty jars when hand warmers and Oxy-Sorb oxygen absorbers were used. To evaluate the effect of hand warmers on hermetic environmental conditions (temperature and relative humidity), we tested 18-h hand warmers (5.08 × 8.89 cm) inside empty 4-gallon glass jars (Figure 1a) and grain-filled 25-kg PICS bags (50 × 85 cm, Figure 1b). The 25-kg PICS bag size was selected because its intergranular air space, when filled with wheat seeds, is approximately equivalent to that of an empty 4-gallon glass jar. The 25-kg PICS bags were sourced from the Purdue University Surplus store (West Lafayette, IN, USA).

Treatments consisted of 2 hermetic environments (empty jars and grain-filled PICS bags) combined with 1, 2, or 3 18-h hand warmers per container. Each treatment was replicated 4 times, totaling 12 empty glass jars and 12 grain-filled PICS bags. Each glass jar was sealed with a rubber airtight lid and then by a second plastic screw lid. A piece of parafilm was wrapped around the second lid to ensure hermeticity. The PICS bags were filled with untreated wheat seeds (variety AG 1189, Alumni Seed Co. Romney, IN, USA). Each layer of the PICS bags was tied separately. All the hand warmers were placed inside the empty 4-gallon jars (Figure 1a) and on top of the grain before sealing each PICS bag (Figure 1b). Two mesh bags filled with 250 g of wheat seeds were placed inside each grain-filled PICS bag with one mesh bag on the top and the other near the bottom of the grain (Figure 1b). The mesh bag at the top was attached to 1 hand warmer to represent the grain in direct contact with hand warmers, while the mesh bag near the bottom represented the grain far from the hand warmers. A jute string was attached to each mesh bag for retrieval.

### 2.3. Data Collection

#### 2.3.1. Cost and Availability of Oxygen Scavengers

To assess the price and availability of hand warmers and Oxy-Sorb oxygen absorbers, we gathered prices from online vendors in the U.S. We compiled a list of websites selling these products and then narrowed it down to vendors with the highest number of sellers and product range.

#### 2.3.2. Oxygen Concentration and Oxygen Depletion

Modifications were made to the hermetic containers to measure oxygen concentration (O_2_%). A fluorescent yellow Oxydot sensor (OxySense Inc., Dallas, TX, USA), used to detect oxygen gradient, was glued near the top and the bottom of each container (Figure 1a,b) using PERMATEX^®^ Clear Adhesive Sealant Silicone RTV 80050 (Solon, OH, USA). An 80 mm circular cut was made in the PICS inner layer, and a 100 mm diameter plastic Petri dish with an Oxydot sensor was attached to this layer using hot glue (Gorilla Glue^®^, Cincinnati, OH, USA). Once the glue was solidified, it sealed the gap between the Petri dish and the inner layer, maintaining hermeticity. An additional 80 mm circle was cut out of the outer woven layer, aligning with the Petri dish on the inner layer, to ensure the Oxydot sensors remained visible [11]. An OxySense^®^ 525OI Oxygen Analyzer (Industrial Physics, Devens, MA, USA) was used to monitor oxygen concentrations within the containers. A fiber optic oxygen reader connected to the OxySense^®^ 525OI measured real-time oxygen concentrations. In the first experiment, measurements were taken at 0, 1, 3, 6, 8, 12, 24, 48, 72, 96, and 120 h. In the second experiment, readings were taken at 0, 1, 3, 6, 8, 12, 24, and 48 h. For the first experiment, oxygen concentration within the 4-gallon (15,200 mL) jars at each time point was converted to oxygen depletion (cc) using Equation (2).

(2) $$Oxygen\;depletion=\left[Ambient\;O2\right]\left(\%\right)\times 15,200\;mL\;–\;\left[residual\;O2\right]\left(\%\right)\times 15,200\;mL$$

where [Ambient O_2_] or [residual O_2_] are the oxygen concentrations. The ambient oxygen concentration was 20.9%.

#### 2.3.3. Temperature and Relative Humidity

To monitor the effects of the hand warmers on the temperature and relative humidity, we used EL-USB-2 (Lascar Electronics Inc., Erie, PA, USA) data loggers. These devices recorded temperature and relative humidity every 30 min. One data logger was kept inside each replicate (Figure 1). Additionally, data loggers were placed inside two empty jars and two grain-filled PICS bags without hand warmers to serve as controls.

#### 2.3.4. Seed Quality Assessment

The moisture content and germination rates of the wheat seeds stored in the PICS bags during the second experiment were measured at the beginning and end to assess the impact of the hand warmer-induced environmental conditions on seed quality. At the beginning, samples were randomly taken from the top, middle, and bottom sections of the grain in each PICS bag and then mixed to obtain a 500 g sample. At the end, the wheat seeds from the mesh bags were analyzed to assess the effect of the hand warmer-induced temperature and relative humidity on moisture content and germination. Moisture content was determined using the oven-dry method [37], where three 15 g replicates from each sample were dried in a tin cup at 103 ± 1 °C for 72 h. The difference in grain weight before and after drying indicated the moisture content. Germination was assessed following the International Rules for Seed Testing [38]. Four 25-seed sub-samples from both initial and post-experiment samples were tested. Seeds were uniformly placed in Petri dishes lined with moist filter paper, kept damp to promote germination, and monitored daily for seven days to evaluate overall seed quality.

### 2.4. Data Analysis

Since both oxygen absorbers were sold in packs of varying quantities, prices were standardized per unit for easier comparison. All statistical analyses were conducted using Prism-GraphPad version 10.0.3. Tukey’s multiple comparison test was used to compare the mean values of oxygen concentration, oxygen depletion, temperature, relative humidity, and moisture content across treatments, with significance determined at a 95% confidence level. Multiple linear regression analysis was performed to evaluate the relationship between oxygen concentration and variables including the type and number of oxygen scavengers, as well as exposure time, first in jars and then in PICS bags. All graphs were made using Microsoft Excel 2016. Oxygen data for Experiment 1 were analyzed for the first 72 h, as oxygen depletion had plateaued by that point.

## 3. Results

### 3.1. Price and Availability of Hand Warmers and Oxy-Sorb Oxygen Absorbers

A 10-h hand warmer, with an average of USD 0.42 ± 0.12 per unit, had prices ranging from USD 0.26 to 0.75 (Table 1). For the 18-h hand warmers, the average unit price was USD 0.89 ± 0.24, with ranges from USD 0.64 to 1.43. The Oxy-Sorb oxygen absorbers had an average price of USD 0.83 ± 0.34 per unit, with prices ranging from USD 0.50 to 1.30. The pricing and unit cost calculation details for each product are available in Appendix A.

### 3.2. Efficacy of Hand Warmers and Oxy-Sorb Oxygen Absorbers

**Figure 2:**
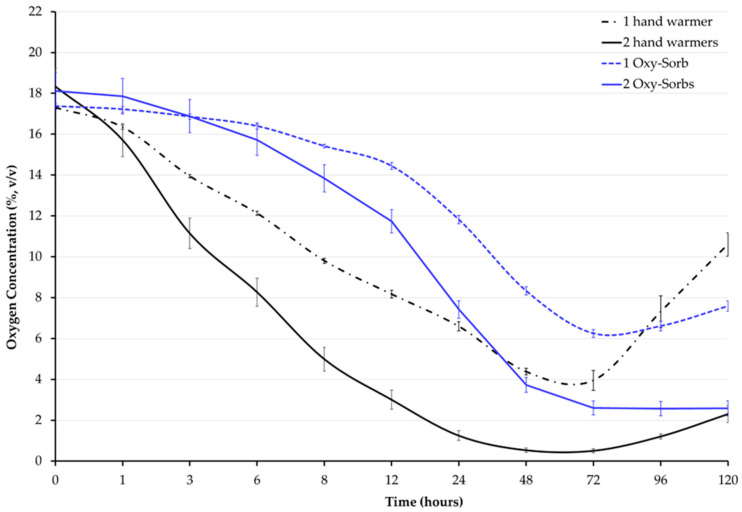
Average oxygen concentration (%) inside empty 4-gallon jars over 120 h. Treatments were 1 or 2 10-h hand warmers or 1 or 2 Oxy-Sorb (2000 cc) oxygen absorbers. Error bars represent standard error of the mean (SEM).

In the first experiment, with one or two 10-h hand warmers or one or two Oxy-Sorb (2000 cc) oxygen absorbers in empty 4-gallon jars, the oxygen concentration (O_2_ %) was affected by treatment (F = 50.48, *p* < 0.001) and time (F = 1819, *p* < 0.001). During the first 48 h, the average oxygen concentration of all treatments, except for the one Oxy-Sorb treatment, dropped below 5% (Table 2 and Figure 2). Only the two 10-h hand warmers treatment reached below 1%. Oxygen concentrations dropped very quickly in the two 10-h hand warmer treatment compared to any of the other treatments. The oxygen concentration ratios for two hand warmers were 0.7 and 0.1 times after 6 and 48 h, respectively, when compared to one hand warmer.

Oxygen depletion (cc) was affected by treatments and time (Table 2). Hand warmers showed faster oxygen depletion than the Oxy-Sorb oxygen absorbers. The two hand warmer treatment exhibited a consistent and significantly higher depletion rate than the other treatments. At 48 h, after reaching their full capacity, there was no significant difference in oxygen depletion between the one hand warmer and two Oxy-Sorb treatments, meaning that they depleted the same amount of oxygen (Table 2). Oxygen depletion rates were faster in hand warmer treatments than with the Oxy-Sorb oxygen absorbers (Appendix B). After 6 h, the oxygen depletion ratios were 1.4, 0.5, and 0.6 times for two hand warmers and one and two Oxy-Sorb oxygen absorbers, respectively, compared to one hand warmer. At 48 h, relative to the plateau value of one hand warmer, the oxygen depletion ratios were 1.2, 0.8, and 1.0 time that for two hand warmers and one and two Oxy-Sorb oxygen absorbers, respectively. The treatments reached their scavenging capacity in a 4-gallon jar at different times: by 24 h for the two hand warmers (2988 ± 37 cc), 48 h for both one hand warmer (2511 ± 25 cc) and two Oxy-Sorb oxygen absorbers (2609 ± 56 cc), and 72 h for one Oxy-Sorb oxygen absorber (2227 ± 30 cc).

A multiple linear regression (Table 3) was estimated for the first 48 h as this period occurred before any treatment reached anoxic conditions. The multiple linear regression had an adjusted R squared of 78% where the effects of the treatment types and the number of units per treatment on oxygen concentration were negative. There was a 2.37 percentage point drop in oxygen concentration per unit time (log_hour) with a unit increase from one to two hand warmers, and a 4.46 percentage point drop in oxygen concentration per unit time (log_hour) when the treatment changed from Oxy-Sorb to hand warmer.

### 3.3. Effect of Hand Warmers on Hermetic Environmental Conditions

**Figure 3:**
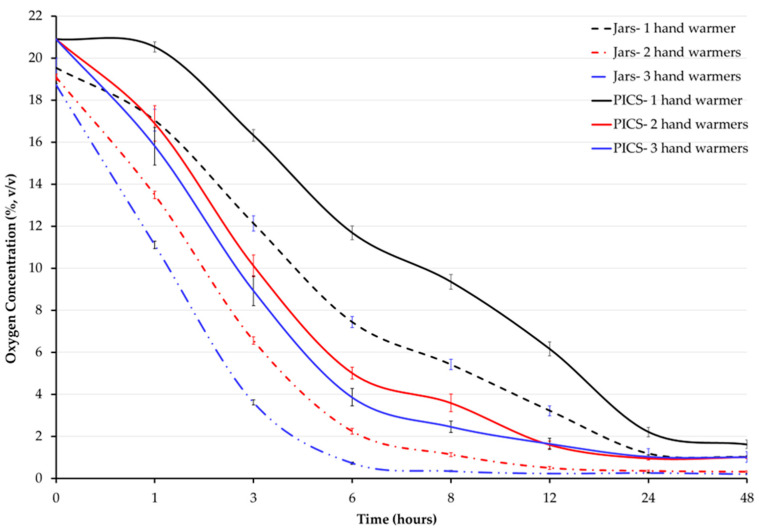
Average oxygen concentration (%) inside empty 4-gallon jars or grain-filled 25-kg PICS bags over 48 h. Treatments were 1, 2, or 3 18-h hand warmers in empty jars or grain-filled PICS bags. Error bars represent standard error of mean (SEM).

In this follow-up experiment using one, two, or three hand warmers in 4-gallon empty jars or grain-filled 25-kg PICS bags, oxygen concentration (O_2_ %) was affected by time (F = 6172, *p* < 0.001) and treatment (F = 107.8, *p* < 0.001), with a significant interaction between time and treatment (F = 53.61, *p* < 0.001). The average oxygen concentration for all treatments, except for the one hand warmer in the grain-filled 25-kg PICS bag treatment, fell below 5% within the first 12 h (Figure 3). Treatments with two or three hand warmers exhibited significantly lower oxygen concentrations compared to the one hand warmer treatment in both the empty jars and the grain-filled 25-kg PICS bags.

Oxygen concentrations decreased rapidly in empty jars but more gradually in PICS bags (Figure 3, Table 4). The fastest decline in oxygen concentration was seen in jars holding three hand warmers, reaching levels below 1% within 6 h. No significant differences were found in the treatments with two or three hand warmers in the PICS bags. In the empty jars, no significant differences were observed between treatments with two or two hand warmers after 12 h.

The storage container type, number of treatment units, and exposure time affected oxygen concentration, with an adjusted R squared of 90% (Table 5). There was a 4.13 and 5.14 percentage point drop in oxygen concentration, respectively, when the number of hand warmers increased from one to two or three. The effect of container type on oxygen concentration was positive, where a grain-filled 25-kg PICS bag had a 2.92 percentage point greater oxygen concentration per unit time (log_hour) than an empty 4-gallon jar.

### 3.4. Temperature and Relative Humidity

**Figure 4:**
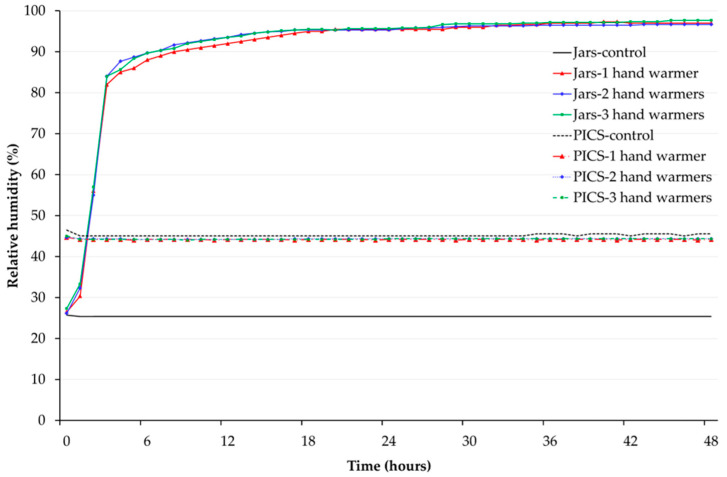
Average relative humidity inside empty 4-gallon jars and grain-filled 25-kg PICS bags over 48 h. Treatments were 1, 2, or 3 18-h hand warmers in empty jars or grain-filled PICS bags.

In the experiment with the empty jars, room temperature was maintained at 21.9 ± 0.29 °C, while for the PICS bag treatments, it was 24.61 ± 0.22 °C. The temperature inside both hermetic storage types increased by less than 2.5 °C and tracked their respective room temperatures regardless of whether one, two, or three hand warmers were used. The average relative humidity in the empty 4-gallon jars over 48 h was constant for the control at 25.34 ± 0.01% but increased asymptotically to near saturation for the one, two, and three hand warmer treatments, reaching 90.83 ± 2.08%, 91.25 ± 2.05%, and 97.01 ± 0.11%, respectively (Figure 4). In contrast, the average relative humidity in the grain-filled 25-kg PICS bags over 48 h was constant and nearly the same for the control and the one, two, and three hand warmer treatments at 45.14 ± 0.04%, 44.13 ± 0.01%, 44.30 ± 0.01%, and 44.27 ± 0.02%, respectively (Figure 4).

### 3.5. Seed Moisture Content and Seed Germination Test

The initial moisture content of the wheat seeds in the PICS bags, measured at 9.76 ± 0.06%, was unaffected by hand warmer treatments (F = 0.07, *p* = 0.933), but exposure time over 48 h did have an impact (F = 6.84, *p* = 0.004). The interaction between treatment and exposure time was not significant (F = 0.27, *p* = 0.89). After 48 h, there was no significant difference in moisture content between the initial seeds and those from the top and bottom layers in the PICS bags, except for in the three hand warmer treatment (Table 6).

The initial seed germination rate was 99%. After 48 h, the seed germination rates from the one, two, and three hand warmer treatments remained equally high at 96%, 97%, and 97%, respectively. No significant differences in germination rates were observed between the initial seeds and those subsequently evaluated from the top and bottom layers in the PICS bags (Table 6).

## 4. Discussion

Our study is the first to explore the use of commercially available hand warmers as oxygen scavengers for enhancing hermetic storage. Results showed that hand warmers were cost-effective and faster than Oxy-Sorb oxygen absorbers in reducing oxygen concentrations within hermetic jars. Quickly achieving low-oxygen environments is crucial to managing insect pests. Research has shown that attaining low oxygen in the first 24 h can profoundly impact insect mortality and progeny development [14,25,39,40]. The impact of the faster oxygen reduction rate of hand warmers compared to Oxy-Sorb oxygen absorbers on insect mortality and progeny development remains to be fully investigated. Increasing the number of hand warmers significantly accelerated oxygen reduction in the hermetic jars, emphasizing the benefit of additional oxygen scavengers and their potential efficacy over Oxy-Sorb oxygen absorbers in the first 48 h. Within 48 h, one hand warmer reduced oxygen concentrations to levels similar to those achieved with two Oxy-Sorb units, indicating that hand warmers are more efficient at lowering oxygen under these hermetic storage conditions. However, some oxygen concentrations started to rebound after 48 h as the oxygen scavengers reached their limits (e.g., chemical capacity), a phenomenon previously observed in hermetic conditions once oxygen drops below 5% [41].

Hand warmers achieved faster and greater oxygen depletion on a per-unit basis and reached their maximum capacity quicker than the Oxy-Sorb oxygen absorbers, highlighting their potential to accelerate oxygen depletion in hermetic storage. Faster oxygen depletion speeds up insect mortality and suppresses progeny development [14,39]. Doubling the number of same-type oxygen scavengers accelerated oxygen depletion but did not yield twice the amount of oxygen depleted. However, both the one hand warmer and two Oxy-Sorb oxygen absorber treatments reached their full capacity simultaneously and depleted similar amounts of oxygen. This finding supports our hypothesis that hand warmers are a viable alternative to conventional oxygen scavengers, such as Oxy-Sorb oxygen absorbers, commonly used in the stored-product industry [19].

The cost per unit for a 10-h hand warmer was roughly half that of a 2000 cc Oxy-Sorb oxygen absorber unit. In addition, since one hand warmer matched the oxygen depletion capacity of three Oxy-Sorb oxygen absorbers in empty jars, we conclude that hand warmers are more cost-effective on a per-unit basis. Beyond cost, availability significantly influences technology adoption among farmers [6]. While Oxy-Sorb absorbers are generally accessible only online, targeting large commercial customers, hand warmers can be found both online and at local retail stores. Many grocery and hardware stores carry hand warmers, although they are typically seasonal items available during colder months. Hand warmers also offer a wide range of pack sizes, from 2 to 240 units, providing flexibility based on storage needs. In contrast, Oxy-Sorb oxygen absorbers were not found in local stores, with online packs limited to quantities of 10, 20, 50, or 200 units. Our findings indicate that HotHands^®^ hand warmers would be preferred over Oxy-Sorb oxygen absorbers due to their faster oxygen depletion rate, lower unit cost, and broader availability, making them more accessible to end users.

Assessing the impact of hand warmers on environmental conditions in hermetic storage is critical, given their composition and mode of action that releases heat and humidity. Therefore, by using one, two, or three hand warmers aimed to evaluate oxygen depletion scalability, assess their effects on temperature and humidity, and provide practical storage recommendations. With the same number of hand warmers and a comparable air volume, oxygen concentrations decreased more quickly in empty jars than in grain-filled PICS bags. While these differences were statistically significant, they would likely be subtle in practical applications. Although hand warmers release heat when activated, temperatures remained constant over 48 h in the empty jars and the grain-filled PICS bags, indicating that heat release should not be a concern when using hand warmers as oxygen scavengers.

The relative humidity in the empty 4-gallon jars increased to near saturation within 24 h, regardless of the number of hand warmers but remained constant in grain-filled 25-kg PICS bags. This increase in relative humidity inside the 4-gallon empty jars is likely due to water evaporation from the hand warmers [29]. In contrast, the observed constant relative humidity in the grain-filled PICS bags probably resulted from grain absorption, as this was the only difference between the two storage environments. Previous studies have shown that the relative humidity in hermetic grain storage remains constant when germinating seeds are used as oxygen scavengers [14].

The effect of the hand warmer treatments on grain moisture content was minimal, with no significant differences observed between the initial and post-exposure moisture levels. However, a notable difference in moisture content was found between the top and bottom of the grain, only in the three hand warmer treatment. These findings suggest that moisture content may rise with an increasing number of hand warmers, highlighting the need for further investigation into moisture dynamics within hermetic storage environments. Based on our current data on seed moisture content and germination rates, we believe that the short-term use of hand warmers during hermetic storage can effectively maintain seed quality. These findings align with previous research showing minimal impact on grain moisture and seed viability when germinating seeds are used as oxygen scavengers [14].

This study explored the potential of hand warmers as oxygen scavengers in hermetic storage. While our results are promising, they also reveal certain limitations that warrant further investigation. Our findings suggest that hand warmers can rapidly reduce oxygen concentration to 5% or below within 48 h, a level lethal to many storage pests. However, achieving this lethal threshold is only one component of effective pest control in hermetic storage; maintaining low oxygen levels over an extended period is equally critical [14,25,39]. Insects can sometimes survive hermetic storage through post-anoxic recovery when not exposed to lethal oxygen levels long enough [42,43]. Research indicates that exposing insects to oxygen levels between 3% and 5% for 4–14 days effectively prevents adult recovery and progeny development [10,25,39]. Future research should monitor oxygen concentrations over short and longer periods, assessing their impact on insect mortality and progeny development to thoroughly evaluate the capacity and effectiveness of hand warmers to control pests in hermetic storage systems.

## 5. Conclusions

This study explored oxygen scavenging in hermetic storage using HotHands^®^ hand warmers as an alternative to commercial Oxy-Sorb oxygen absorbers. Findings suggest that hand warmers can effectively and rapidly deplete oxygen to lethal levels for stored-product insects, with minimal impact on environmental conditions and grain quality in hermetic storage. Hand warmers offer several advantages over Oxy-Sorb oxygen scavengers. A single hand warmer is more cost-effective than an Oxy-Sorb absorber, costing half as much while depleting twice the oxygen volume. Moreover, using three hand warmers instead of two offers minimal benefits in reducing the time required to reach the 5% oxygen threshold for pest suppression. Widely available in retail stores, hand warmers provide farmers with a practical, chemical-free solution for storing specialty crops or small quantities of seeds or grain. However, further research is needed to assess the effectiveness of hypoxia induced by hand warmers for both short- and long-term storage and its impact on pest control and grain quality.

While hand warmers show potential for improving hermetic storage, they face several challenges, including limited oxygen absorption capacity, which restricts their effectiveness in larger hermetic containers, and the generation of humidity, which could compromise grain quality. Moreover, their availability is largely confined to countries with winter climates, limiting their use in tropical regions where they may be most needed. Since their use is currently better suited for small hermetic storage containers (e.g., jars, bags, drums, and small silos), scaling up would require broader availability, particularly in tropical regions, enhanced capacity (e.g., nanotechnology), targeting high value crops or products, and addressing humidity release through design improvements.

## Figures and Tables

**Table 1:** Capacity and average price and range of HotHands^®^ hand warmers and Oxy-Sorb oxygen absorbers. Prices were collected from Amazon and Walmart online stores on 20 May 2024.

Products	Capacity	Average Price per Unit (USD)	Price Range per Unit (USD)
HotHands^®^ Hand warmer	10-h	0.42 ± 0.12 (n * = 24)	0.26–0.75
HotHands^®^ Hand warmer	18-h	0.89 ± 0.24 (n = 16)	0.64–1.43
Oxy-Sorb oxygen absorber	2000 cc	0.83 ± 0.34 (n = 7)	0.50–1.30

* n = total number of sellers among online vendors. More details on prices of packs and vendors are in Appendix A.

**Table 2:** Average oxygen concentration (%) and depletion (cc) in empty 4-gallon jars over 72 h. Treatments were 1 or 2 10-h hand warmers or 1 or 2 Oxy-Sorb (2000 cc) oxygen absorbers in empty 4-gallon jars.

			Oxygen Concentration (%, Mean ± SEM) *				
				Time			
Variable	Treatment	0 h	6 h	12 h	24 h	48 h	72 h
Oxygen concentration (%)	1 hand warmer	17.3 ± 0.09 aA	12.1 ± 0.09 bB	8.2 ± 0.19 cC	6.6 ± 0.22 bD	4.4 ± 0.17 bE	4.0 ± 0.49 bE
	2 hand warmers	18.3 ± 0.91 aA	8.3 ± 0.68 cB	3.0 ± 0.46 dC	1.3 ± 0.24 cDE	0.5 ± 0.11 cE	0.5 ± 0.10 cE
	1 Oxy-Sorb	17.5 ± 0.13 aA	16.4 ± 0.15 aA	14.4 ± 0.17 aB	11.8 ± 0.20 aC	8.3 ± 0.19 aD	6.3 ± 0.20 aE
	2 Oxy-Sorbs	18.1 ± 0.91 aA	15.7 ± 0.75 aB	11.7 ± 0.58 bC	7.4 ± 0.42 bD	3.7 ± 0.37 bE	2.6 ± 0.34 bE
Oxygen depletion (cc)	1 hand warmer	547 ± 13 aE	1333 ± 14 bD	1935 ± 28 bC	2174 ± 34 bB	2511 ± 25 bA	2576 ± 75 bA
	2 hand warmers	389 ± 138 aD	1920 ± 103 aC	2720 ± 70 aB	2988 ± 37 aA	3096 ± 17 aA	3100 ± 15 aA
	1 Oxy-Sorb	537 ± 20 aE	684 ± 24 cE	982 ± 26 dD	1381 ± 31 cC	1910 ± 30 cB	2227 ± 30 cA
	2 Oxy-Sorbs	422 ± 138 aE	786 ± 115 cD	1392 ± 88 cC	2049 ± 64 bB	2609 ± 56 bA	2781 ± 51 bA

* All the data are means ± the standard error of means (SEM). Under the same variable, within the same time, or among treatments (lowercase), or within each treatment or across times (uppercase), means followed by the same letter are not significantly different (*p* < 0.05).

**Table 3:** The multiple regression of oxygen concentration (%) as a function of the number of oxygen scavenger units (1 or 2), type of oxygen scavenger (Oxy-Sorb or hand warmer), and time (log_hour), with estimated marginal slopes, standard error, t, and *p* values. The treatments were of 1 or 2 10-h hand warmers or 1 or 2 Oxy-Sorb (2000 cc) oxygen absorbers in empty 4-gallon jars.

Variable	Estimated Marginal Slopes	Standard Error	t Value	*p*-Value
Intercept	18.58	0.44	42.18	<0.0001
1 unit	0.00	0.00	.	.
2 units	−2.37	0.46	5.18	<0.0001
Oxy-Sorb	0.00	0.00	.	.
Hand warmer	−4.46	0.46	9.72	<0.0001
Time (log_hour)	−5.20	0.29	17.99	<0.0001

**Table 4:** Average oxygen concentration (%) inside empty 4-gallon jars and grain-filled 25-kg PICS bags over 48 h. Treatments were 1, 2, or 3 18-h hand warmers in empty jars or grain-filled PICS bags.

	Oxygen Concentration (%, Mean ± SEM) *					
	Time					
Treatment	0 h	3 h	6 h	12 h	24 h	48 h
Jar-1 Handwarmer	19.6 ± 0.45 bA	12.1 ± 0.36 bB	7.4 ± 0.27 bC	3.2 ± 0.24 bD	1.2 ± 0.24 abE	1.0 ± 0.25 abE
Jar-2 Hand warmers	19.1 ± 0.15 bA	6.6 ± 0.18 dB	2.2 ± 0.14 dC	0.5 ± 0.07 deD	0.4 ± 0.04 bD	0.3 ± 0.05 bD
Jar-3 Hand warmers	18.7 ± 0.06 bA	3.6 ± 0.12 eB	0.7 ± 0.05 eC	0.2 ± 0.03 eC	0.3 ± 0.03 bC	0.2 ± 0.04 bC
PICS-1 Hand warmer	20.9 ± 0.00 aA	16.3 ± 0.25 aB	11.7 ± 0.33 aC	6.2 ± 0.34 aD	2.2 ± 0.22 aE	1.6 ± 0.21 aE
PICS-2 Hand warmers	20.9 ± 0.00 aA	10.1 ± 0.51 cB	5.0 ± 0.28 cC	1.6 ± 0.18 cdD	1.0 ± 0.08 abD	1.0 ± 0.06 abD
PICS-3 Hand warmers	20.9 ± 0.00 aA	8.9 ± 0.71 cB	3.9 ± 0.41 cC	1.7 ± 0.26 cdD	1.0 ± 0.09 abD	1.0 ± 0.05 abD

* All data are the means ± the standard error of means (SEM). Within the same time point (lower case) or within the same treatment (upper case), the means followed by the same letters are not significantly different (*p* > 0.05).

**Table 5:** Multiple regression of oxygen concentration (%) as a function of number of hand warmer units (1, 2, or 3), type of hermetic containers (empty 4-gallon jars or grain-filled 25-kg PICS bags), and time (log_hour), with estimated marginal slopes, standard error, t, and *p* values. Treatments were 1, 2, or 3 18-h hand warmers in empty jars or grain-filled PICS bags.

Variable	Estimated Marginal Slopes	Standard Error	t Value	*p*-Value
Intercept	14.67	0.24	59.56	<0.001
1 Hand warmer	0.00	0.00	.	.
2 Hand warmers	−4.13	0.26	15.89	<0.001
3 Hand warmers	−5.14	0.26	19.80	<0.001
Jars	0.00	0.00	.	.
PICS	2.92	0.21	13.77	<0.001
Time (log_hour)	−9.03	0.17	51.58	<0.001

**Table 6:** Average (±SEM, n = 4) moisture content and germination rate of wheat seeds from initial measurements and 48 h later (top and bottom sample bags). Treatments were 1, 2, or 3 18-h hand warmers in empty 4-gallon jars or grain-filled 25-kg PICS bags.

Variable	Treatment *	Initial	Opening Time: After 48 h	
			Top	Bottom
Seed moisture content (%) *	1 hand warmer	9.76 ± 0.06 aA	10.21 ± 0.78 aA	9.63 ± 0.01 aA
	2 hand warmers	9.76 ± 0.06 aA	10.47 ± 0.17 aA	9.56 ± 0.28 aA
	3 hand warmers	9.76 ± 0.06 aA	10.66 ± 0.28 aA	9.45 ± 0.29 aB
Seed germination rate (%) *	1 hand warmer	99.00 ± 1.00 aA	96.00 ± 1.25 aA	96.00 ± 1.00 aA
	2 hand warmers	99.00 ± 1.00 aA	97.00 ± 1.00 aA	97.00 ± 1.25 aA
	3 hand warmers	99.00 ± 1.00 aA	97.00 ± 1.25 aA	97.00 ± 1.25 aA

* Within the same variable, under the same time point, means within the same treatment (lowercase letter) and the same row (uppercase letter) followed by the same letter are not significantly different (*p* > 0.05).

## Data Availability

The original contributions presented in the study are included in the article, further inquiries can be directed to the corresponding author.

## Appendix A

**Table A1:** Price comparison of oxygen scavenger products used in this study sourced from Amazon and Walmart as of 20 May 2024.

Hothand^®^ 10-h Hand Warmer				Hothand^®^ 18-h Hand Warmer			
Supplier	Number per Pack	Price (USD)	Price per Unit (USD)	Supplier	Number per Pack	Price (USD)	Price per Unit (USD)
Amazon	10	5.75	0.58	Amazon	1	2.74	0.27
	20	9.99	0.50		3	4.79	1.60
	20	7.48	0.37		3	3.22	1.07
	40	29.80	0.75		4	6.99	1.75
	40	15.01	0.38		8	7.63	0.95
	60	24.95	0.42		10	10.99	1.10
	60	22.99	0.38		10	8.92	0.89
	80	25.05	0.31		20	14.99	0.75
	80	21.18	0.26		40	27.39	0.68
	100	30.98	0.31	Walmart	3	4.82	1.61
	108	34.99	0.32		5	8.03	1.61
	200	66.25	0.33		6	8.60	1.43
	200	59.95	0.30		10	7.48	0.75
	240	81.84	0.34		40	25.99	0.65
					54	34.74	0.64
Walmart	2	0.98	0.49		120	109.57	0.91
	10	5.75	0.58	**Oxy-Sorb (2000 cc) oxygen absorbers**			
	20	9.99	0.50	Supplier	number per pack	Price (USD)	Price per unit (USD)
	20	7.48	0.37	Amazon	10	12.99	1.30
	24	15.98	0.67		20	10.51	0.53
	40	16.26	0.41		30	15.00	0.50
	60	29.99	0.50	Walmart	10	12.99	1.30
	80	25.99	0.32		20	12.99	0.65
	100	33.99	0.34		30	24.99	0.83
	108	36.90	0.34		50	35.00	0.70

## Appendix B

**Table A2:** Oxygen depletion rates (cc) in empty 4-gallon jars over 120 h and depletion ratios based on 1 hand warmer. Different letters represent treatments consisted of 1 or 2 10-h hand warmers or of 1 or 2 units of Oxy-Sorb (2000 cc) oxygen absorbers in empty 4-gallon jars.

Time	Oxygen Depletion Rates (cc per Hour)				Depletion Ratio Based on 1 Hand Warmer			
	1 Hand Warmer	2 Hand Warmers	1 Oxy-Sorb	2 Oxy-Sorbs	1 Hand Warmer	2 Hand Warmers	1 Oxy-Sorb	2 Oxy-Sorbs
1	691.98 ± 15 a	788.12 ± 123 a	559.36 ± 21 b	461.32 ± 132 b	1	1.14	0.81	0.67
3	352.37 ± 4 a	494.38 ± 38 a	204.82 ± 7 b	203.68 ± 41 b	1	1.41	0.58	0.58
6	222.17 ± 2 a	320.02 ± 17 a	114.06 ± 4 b	131.04 ± 19 b	1	1.44	0.51	0.59
8	210.81 ± 3 a	302.29 ± 11 a	104.17 ± 2 b	134.19 ± 13 b	1	1.43	0.49	0.64
12	161.25 ± 2 b	226.67 ± 6 a	81.80 ± 2 d	1160 ± 7 c	1	1.41	0.51	0.72
24	90.60 ± 1 b	124.48 ± 2 a	57.52 ± 1 c	85.36 ± 3 b	1	1.37	0.64	0.94
48	52.32 ± 1 b	64.50 ± 0 b	39.80 ± 1 b	54.36 ± 1 b	1	1.23	0.76	1.04
72	35.78 ± 1 b	43.06 ± 0 a	30.93 ± 0 c	38.63 ± 1 b	1	1.20	0.86	1.08
96	21.52 ± 1 b	31.18 ± 0 a	22.63 ± 0 b	29.02 ± 1 a	1	1.45	1.05	1.35
120	13.04 ± 1 b	23.56 ± 1 a	16.87 ± 0 b	23.20 ± 0 a	1	1.81	1.29	1.78
